# Do as I do imitation in a steller sea lion *Eumetopias jubatus*

**DOI:** 10.1007/s10071-025-01971-0

**Published:** 2025-06-20

**Authors:** Masahiro Sasaki, Hinano Kinoshita, Akitsugu Konno

**Affiliations:** 1 Kinosaki Marine World, 1090 Seto, Toyooka, Hyogo 669-6192 Japan; 2 Department of Animal Science and Biotechnology, Azabu University, 1-17-71 Fuchinobe, Chuo-ku, Sagamihara City, Kanagawa 252-5201 Japan

**Keywords:** Pinnipeds, Social learning, Do as I do, Training, Steller sea lions, Motor imitation

## Abstract

The present study explored whether a well-socialized Steller sea lion named Hama could reproduce similar actions with human demonstrations using the “Do as I do” (DAID) paradigm. Hama had learned 50 types of behaviors, but her social learning ability was unknown. In Study 1, we trained Hama to produce simultaneous DAID responses. After introducing the DAID training, we conducted four tests to confirm Hama’s acquisition of demonstrator-matching behavior. We found that Hama successfully acquired the action-matching ability for human actions, not only with three trained actions but also with six out of seven untrained actions (see Test 1 and Test 2). Moreover, Hama’s DAID performance was stable regardless of the familiarity of human demonstrators (see Test 1 and Test 2). Hama successfully performed two completely novel body actions that were not included in her prior learning repertoire, but failed to replicate actions involving object manipulation (see Test 3). She showed no response in control trials without demonstrations, providing partial evidence for negative control (see Test 4). In Study 2, we introduced Hama to performing non-simultaneous DAID responses, which involved suppressing immediate simultaneous actions following the demonstrator and then reproducing the actions upon the verbal cue “Go.” She accurately performed the DAID response even when she delayed her response until the demonstrator’s demonstration was completed (Test 5). Importantly, she reproduced the action accurately when visual contact between the demonstrator and herself was blocked after the demonstration, eliminating the Clever Hans effect as a potential influence on her simultaneous DAID response (Test 6). However, she could not reproduce small or untrained actions (Test 7). These results suggest that Hama may be able to accurately map human action sequences in body-oriented actions to some extent. This study provides the first evidence of motor imitation ability in captive pinnipeds.

**Supplementary Information:**

The online version contains supplementary material available at 10.1007/s10071-025-01971-0.

## Introduction

Social learning, also known as observational learning, plays an important role in the evolution and development of behaviors in many social species (Schaik and Burcart 2011). Observers acquire knowledge about food sources, habitat environment, potential predators, and social partners by watching the behaviors of conspecifics (same species) and heterospecifics (different species) (Webster 2023). This social cognitive ability confers adaptive advantages on individuals especially in group living animals (Ward and Webster 2016). Social learning can take many forms, including local (or stimulus) enhancement, emulation, and motor imitation (Whiten et al. 2004; Lea and Osthaus 2018).

The “Do as I Do (DAID)” paradigm (Topal et al. 2006) is an experimental procedure used to assess motor imitation of human actions in captive animals. During DAID training, animals are conditioned to associate a small set of their own actions with human actions following a specific vocal cue, such as "Do it!" The test phase evaluates whether subjects can select a functionally similar behavior in response to the actions demonstrated by the human. Subjects were then tested with an unfamiliar demonstrator or with untrained actions (Fugazza and Miklosi 2015). Transfer of motor imitation to these novel stimuli suggests that the subjects have acquired the basic rule to reproduce similar behaviors based on observing human actions (Zentall 2012).

The DAID paradigm originated from a study with captive chimpanzees *Pan troglodytes* (Hayes and Hayes 1952). Subsequent studies using this procedure have revealed that it can induce motor imitative behaviors in great apes (Myowa-Yamakoshi and Matsuzawa 1999; Call 2001). Dogs *Canis familiaris* are currently the most extensively studied animals in terms of DAID imitation. Early studies examined the imitation skills of two dogs (Philip and Joy), separately, and found that dogs had the potential to generalize their DAID response to not only trained actions but also to novel ones (Topál et al. 2006; Huber et al. 2009). Later studies offered evidence that DAID training facilitates dogs' imitative behavior even with a delay after the human demonstration, suggesting the presence of deferred imitation in dogs (Fugazza and Miklósi 2014; Fugazza and Miklósi 2015; Fugazza et al. 2016).

On the other hand, DAID research on other species is limited. There have been reported successful cases of DAID training with highly-socialized companion animals, including cats *Felis catus* (Fugazza et al. 2020) and grey parrots *Psittacus erithacus* (Moore 1993; Pepperberg 1988). Studies with captive cetaceans also suggest that bottlenose dolphins *Tursiops truncatus*, orcas *Orcinus orca*, and belugas *Delphinapterus leucas* can potentially imitate human or conspecific actions after DAID training (Herman et al. 2001; Herman 2002; Zamorano-Abramson et al. 2012, 2017, 2018, 2023). As Zamorano-Abramson et al. (2023) summarized, while most studied species could be trained for familiar actions using DAID imitation, only chimpanzees, dogs, dolphins, and orcas showed transfer of this behavior to novel situations. Comparative studies including animals from diverse phylogenetic groups would be informative to understand the evolutionary constraints of the cognitive ability to form a generalized behavior-copying concept.

We focused on Steller sea lions *Eumetopias jubatus*, the largest and one of the most widespread species within the family of sea lions (Otariidae) belonging to Pinnipeds (Gentry 1970). The Steller sea lions are polygynous, forming reproductive rookeries of dozens to hundreds of individuals during the breeding season. Wild populations of the species exhibit various social behaviors, including “boundary display”, “neck bite”, and “open mouth submissions," to maintain social relationships and spatial territories (Gentry 1974). They can produce a range of vocalizations used in communication between mothers and pups, as well as between males and females (Louring 2009). A closely related species with Steller sea lions, California sea lions *Zalophus californianus*, have found that knowledge about novel feeding sites was transferred to other group members via social networks, suggesting evidence of social learning in Otariidae in their natural habitat (Schakner et al. 2017).

Sea lions in captivity can be trained to respond to various action signals or vocal signals by human trainers (Kastak and Schusterman 1994; Sasaki et al. 2022). This makes them a fascinating animal model for examining the cognitive traits regarding semantic or symbolic comprehension. California sea lions have been successfully tested using a generalized identity matching-to-sample paradigm. A female subject, Rio, has even acquired functional stimulus equivalence, including demonstrating symmetric, transitive, and equivalent relations between visual stimulus pairs (Schusterman and Kastak 1998, 1993). Recently, Sasaki and Kambara (2024) found that a Steller sea lion can respond to a combination of two different verbal commands from humans, effectively selecting another learned behavior with similar body parts and action sequences. These findings suggest that Otariidae possess competence for abstraction and conceptual learning, elicited by semantic training by humans. While the cognitive traits of sea lions have been examined, research on social learning in these animals remains in its early stages.

This study aimed to examine whether Steller sea lions could develop social skills for observing and imitating human actions. We trained Hama, a captive Steller sea lion with a repertoire of 50 learned actions, using a DAID paradigm based on a dog study (Topál et al. 2006). To assess the effect of DAID training on Hama’s action choices, we conducted two studies. Study 1 examined simultaneous DAID responses, consistent with Hama's prior training to respond immediately to human instructions. First, we evaluated if Hama had learned the DAID responses for the three trained actions (Test 1). Second, we examined if Hama could apply these responses to seven untrained actions (Test 2). To rule out the possibility of unintentional cues from the familiar trainer, we tested Hama's performance with a new, unfamiliar trainer for both trained (Test 1) and untrained actions (Test 2). Third, we tested Hama's ability to copy completely novel actions she had never learned before (Test 3). Additionally, we included "No demonstration" trials (Test 4) as a negative control to confirm that Hama's responses were not due to non-specific or biased action selection.

In Study 2, we tested Hama’s ability to perform DAID responses with a delay following the completion of the human demonstration and the generic “Go” verbal command, assessing her capacity for non-simultaneous imitation using a standard DAID paradigm (Topal et al. 2006). This paradigm, where subjects select human actions after a generic voice command (e.g., “Do it!”), has been used in previous studies on dogs (Fugazza and Miklósi 2014; Fugazza and Miklósi 2015; Fugazza et al. 2016). Killer whales have also been studied in detail to determine their ability to perform the DAID response correctly, even with a delay after the human demonstration. This line of research aims to rule out simpler learning mechanisms other than imitation in the DAID response (Zamorano-Abramson et al. 2023). Successful non-simultaneous DAID performance by Hama would demonstrate strong motor imitation capabilities for the following two key reasons. First, it requires her to rely on memory representations to select and execute the action, rather than simply reacting to the demonstrator's movement. Second, the delay allows for visual separation, effectively ruling out the influence of unintentional movement cues from the demonstrator. We then conducted three tests: (1) to evaluate whether Hama had learned the non-simultaneous responses for the four trained actions (Test 5), (2) to examine the test with a partition blocking visual contact between Hama and the demonstrator after the demonstration (Test 6), and (3) to examine whether Hama could apply non-simultaneous DAID to four untrained actions (Test 7).

In summary, this study investigates the motor imitation ability of a sea lion (Hama) with experience learning over 50 actions, using both simultaneous and non-simultaneous DAID paradigms. Notably, the current work would be the first case study to document demonstrator-matching behavior in pinnipeds.

## Methods

### Subject

The subject was a female Steller sea lion, Hama, who was 13 years old at the beginning of this study. Hama learned 50 types of actions associating with human vocal commands and hand signals. We trained all actions by operant conditioning, and vocal commands and hand signals were conditioned secondarily after shaping each action. She has been reinforced to respond immediately to human-given cues in all her training history. We have never used imitative methods (e.g. using demonstration) to generate her actions. Details on how Hama was kept and trained can refer to Sasaki et al. (2022), and details on learned behaviors and commands can refer to Sasaki and Kambara (2024).

### Experimenter

Because of the animal's characteristics, it is difficult for a completely new person to interact with Hama, we selected trainers who are engaged in the daily care of Hama as experimenters. The experimenters were one male trainer who conducted “DAID training” to Hama (experimenter: MS) and one female trainer who was not involved in the DAID training (novel experimenter: HK).

### Defining key terms for target actions

Throughout this paper, we will use two key terms to describe the target actions. First, "untrained" refers to actions that Hama has already been trained to perform (meaning they are in her existing learning repertoire) but for which she has not received specific training within the DAID paradigm; this was verified in Test 2 and Test 7. Second, "(completely) novel" refers to actions that Hama has never been trained to perform and are thus not in her learning repertoire; these were verified in Test 3.

### Study 1: simultaneous DAID response in Hama

This experiment was based on the DAID method by Topal et al. (2006) but partially modified as follows.

i) Choice of action based on Hama’s learning history

The actions Hama had already learned were conditioned with hand signals or vocal commands given by the trainers. These learned actions were reinforced entirely by operant conditioning, and they were never associated with demonstrations of action by the trainers. In this experiment, we selected target actions from Hama’s learned actions in which (1) the human demonstrator could produce similar actions in terms of body parts and behavioral sequence and (2) Hama’s action and trainer’s signals were physically different (e.g., When the trainer held up an index finger, Hama sticked out her tongue). In contrast, we excluded the learned actions that the trainer's hand signal and Hama's actions were similar (e.g., when the trainer waved his hand, Hama waved her fore flipper). The above criteria were met by 10 types of actions in Hama’s learning repertoire (see Table 1). Vocal commands and hand signals that indicate these actions can be viewed at https://youtu.be/-Kwv_fMeG5w.

**Table 1:** Definition of action types demonstrated by the experimenters and the expected actions produced by Hama during the DAID training and testing

Label	Experimenter’s demonstration of actions	Hama's expected “correct” action for full correspondence
*Test 1: The trained actions*		
Stand	E stands up from taking kneel down	H stands up and place its fore flippers on the cage
Head	E shakes head sideways several times	H shakes head left and right several times
Tongue	E sticks one's tongue out of the mouth	H sticks her tongue out of the mouth
*Test 2: the untrained actions*		
Mouth	E opens one's mouth	H opens her mouth
Nod	E moves one's head up and down several times	H moves her head up and down several times
Spin	E rotating once counterclockwise	rotating once clockwise
Down	E lowers one's head and lies down on the ground	H lowers hers head and lies down on the ground
Back	E lies on one's back	H lies on her back
Leg	E swings one's legs several times	H swings her hind flippers several times, either in a prone position or in a handstand position
Kiss	E puts one's right hand over mouth and then release it	H puts her left fore flipper over her muzzle and then release it
*Test 3: The novel actions not in the learning repertoire*		
Hand	E raises one's left hand and holding it still	H raises her right fore flipper and holding it still
Down-Oppo	E lies on one’s face left side	H lies on her head right side
Ball + Face	E touches the ball with one's face	H touches the ball with her muzzle
Ball + Hand	E touches the ball with one's hand	H touches the ball with her fore flipper
Fin + Face	E touches the fin with one's face	H touches the fin with her muzzle
Fin + Hand	E touches the fin with one's hand	H touches the fin with her fore flipper
Hose + Face	E touches the hose with one's face	H touches the hose with her muzzle
Hose + Hand	E touches the hose with one's hand	H touches the hose with her fore flipper
*Test 4: no demonstration*		
No	E do not demonstrate any action	H remains passive position and does not perform any action types

Since the demonstration was performed with the experimenter facing Hama, the direction of movement and the forelimbs to be moved were defined to be opposite for Spin, Down, and Kiss

Topal et al. (2006) divided target actions into "body-oriented" and "object-manipulative" behaviors. However, we focused mainly on body-oriented behaviors during initial phase of DAID training because Hama was not familiar with novel artificial objects in her housing environment and had no prior experience in learning object manipulation behaviors. We also excluded actions to move to a specific location because training site was limited in space (i.e., due to the trainer and Hama being separated by a cage for safety reasons).

ii) Use of simultaneous DAID response

In this study, we intentionally avoided using the vocal cue "Do it!" to signal the start of the subject’s imitation response. Instead, we allowed Hama to respond immediately after the trainer’s demonstration. This decision was based on the following reasons. First, Hama had a long history (nearly 10 years) of being reinforced for simultaneous responses to trainer-given action cues, which prevented Hama from learning delayed responses. Second, our attempts to teach Hama non-simultaneous responses using so-called Wait & Go training were unsuccessful, leading to her reluctance to participate in training. To encourage Hama to exhibit more natural and spontaneous imitation behavior, we chose to allow simultaneous responses. Instead, we used experimental location and a specific action command as cues for the start of the DAID session. In each session, experimenters transferred Hama to a 10.3 m^2^ (4.3 m × 2.4 m) cage that was not usually used as training. Moreover, an experimenter pointed twice alternately to Hama and the experimenter just before the start of session, which were conditioned as cues for the start of the DAID session. Data coding methods are documented in detail in the “Data analysis” chapter.

iii) Use of positive reinforcement in all test sessions

In Topal et al. (2006), the test phase was conducted without reinforcement. However, testing without reinforcement could have decreased Hama's motivation to participate in the test session. Given Hama’s learning history of receiving food rewards for correct responses in previous training, we decided to reinforce all of her responses during all test sessions (i.e., Test 1 to Test 4). Positive reinforcement in all test trials might have influenced Hama’s performance, potentially creating a learning effect. To assess this, we analyzed whether Hama’s ‘first’ response matched the human demonstration and if the “correct” responses increased over trials (see chapter on “Data analysis” for details).

#### Do as I do training phase

Out of 10 targeted action types in Hama’s learning repertoire, we selected three actions for the initial training phase: "Stand," "Head," and "Tongue". Definitions of the ideal “correct” response of Hama corresponding to each human action were shown in Table 1.

**Fig. 1:**
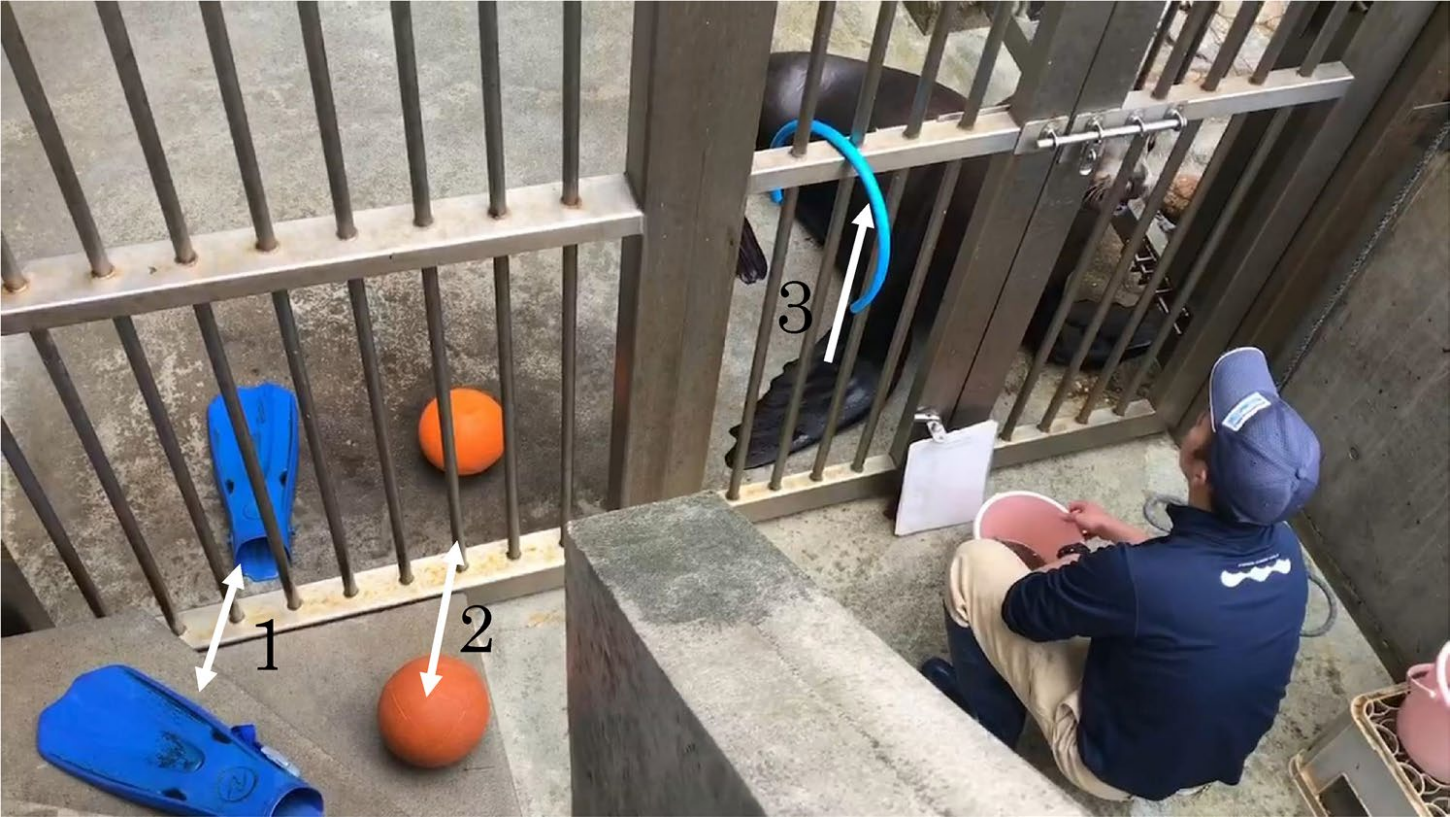
Arrangement of the materials in the experimental area in Test 3. (Note: Arrow 1 indicates swim fins; arrow 2, balls; and arrow 3, a hose)

The training session was conducted with Hama inside a cage alone and the trainer facing Hama outside the cage (Fig. 1). After giving the particular action signals of the DAID session, the experimenter (MS) demonstrated each action and observed Hama’s actions, reinforcing the actions with food reward only when the expected actions were observed. When the expected actions were not produced solely by showing the trainer’s action, experimenter (MS) induced the expected actions by simultaneously giving vocal commands or hand signals that Hama had already learned. The demonstration of these learned cues was gradually reduced so that the respective actions were produced solely by the trainer’s action (i.e., prompt fading). However, the use of learned cues was kept to a minimum frequency, which would facilitate that Hama’s associative learning between the trainer's actions and her own actions accordingly.

For each action, we began training on October 12, 2023, for about 5 min per session. The expected actions were successfully elicited in the second session for the "Stand" command, and in the fourth session for the "Head" and "Tongue" commands, respectively. Thereafter, a total of 20 sessions of DAID training was conducted irregularly until October 30.

#### Do as I do test phase

During the test sessions, we only presented action demonstrations and never provided any prompts. Hama received a food reward (i.e., a piece of fish) for all of her responses during test sessions. Hama was sensitive to the absence of food rewards (as noted above) but did not appear to be sensitive to the quality of the food. Due to the variety of fish available, we could not provide the same type of food reward in every trial. To ensure that the quality of the reward did not affect her responses, we deliberately randomized the type of fish given in each trial (i.e., we ensured that there was no bias in the types of rewards given, regardless of the action type).

To eliminate the possibility of unconscious cueing (i.e. also known as the Clever Hans effect) from the demonstrator, the initial positions, postures (i.e., crouching down and making eye contact with Hama), and distances between the experimenter and Hama were kept consistent across all trials. Additionally, in Test 1 and Test 2, control tests were conducted by a novel experimenter (HK) who had no prior involvement in basic DAID training. This approach closely followed the control condition used by Topal et al. (2006). The detailed procedure for each test is as follows.

i) Test 1: The trained actions

To evaluate the learning outcome of the three actions acquired through the DAID method, a match to sample test was conducted with the experimenter (MS) acting as the demonstrator. We tested a total of 30 trials, with 10 trials for each of three action type (i.e., "Standing" "Head" and "Tongue"). Each test session consisted of six trials in random order, with two trials per action type. We conducted a total of five sessions on November 1, 2022, and November 2, 2023. During the test session,

To control for the possibility of unconscious cueing from the experimenter, a novel experimenter (HK) then tested Hama under identical conditions. The test procedure was the same as when the experimenter (MS) conducted the test, but the presentation order of the actions was randomized. Tests were conducted two sessions on November 1, 2023, and three sessions on November 2, 2023.

ii) Test 2: The untrained actions

After testing the three actions learned through the DAID method, we proceeded to test untrained actions. The target behaviors consisted of seven actions that could be replicated by humans. All of these “untrained actions” were already part of Hama’s learning repertoire. However, none of these actions had been trained through the DAID method. For these actions, we defined in advance the actions of Hama that corresponded to those of the demonstrator (Table 1).

The experimenter (MS) served as the demonstrator for the match-to-sample test. We tested a total of 100 trials, with 10 trials for each of ten action type. Each session consisted of 10 trials including seven untrained and three trained actions. We conducted a total of 10 sessions from November 15 to November 28, 2023. To minimize potential learning effects, test session was conducted only once per day. The trial order was determined by another researcher (one of the authors of this study) who was not familiar with Hama. To help Hama understand that these tests were DAID sessions, the first action in each test session was chosen from the trained actions. In addition, to reduce Hama’s cognitive load caused by the succession of novel stimuli, no more than three untrained actions were presented consecutively.

As a control condition similar to Test 1, the novel experimenter (HK) conducted the same a match-to-sample test as the demonstrator from December 1 to December 19, 2023. The testing method was the same as described above, but the order in which each action was presented was determined anew.

iii) Test 3: The novel actions not in the learning repertoire

As shown in the results, Hama successfully matched her actions with those of the human demonstrator, even in untrained actions. To further explore Hama’s ability to produce similar action corresponding to those given by humans, we examined her responses to completely novel actions that had never been reinforced before and were not included in her prior learning repertoire. In this test, we targeted a total of eight novel actions, including not only two body-oriented actions (i.e., Hand and Down-Oppo) but also six object-manipulation ones (i.e., Ball-Face, Ball-Hand, Fin-Face, Fin-Hand, Hose-Face, and Hose-Hand) (Table 1).

Since Hama had no prior experience with object manipulation training, we acclimated her to the objects used in Test 3. For one month before the test, we placed three objects (i.e., a ball, a fin, and a hose) in her cage for approximately five min each day, allowing her to freely interact with them during feeding time. As a result of acclimation, Hama is no longer averse to contact with each object. The purpose of Test 3 was to determine if Hama could reproduce actions she had never learned before, based solely on human demonstrations. Therefore, we did not conduct any additional training to teach her how to manipulate each object.

During the test session, we placed three objects (i.e., a ball, a fin, and a hose) on the ground always in the same arrangement (see Fig. 1). A total of six action types was determined based on the combination of three objects and two “touch” movements with either hand or face of a human. Only the first experimenter (MS) served as the demonstrator.

In test sessions, we conducted a total of 99 trials, with 10 trials for each of 10 action type including eight novel and two previously tested actions (i.e., the “Kiss” command was only performed nine trials due to a procedural error). Each test session consisted of 10 trials and was conducted only once per day. We conducted a total  of 10 sessions from March 26 to April 10, 2024. The trial order was determined in advance and randomized. Each test session began with two learned actions in Test 2 (i.e., Spin and Kiss), followed by eight novel actions.

iv) Test 4: No demonstration trials (negative control)

To confirm that Hama's responses were based on the pre-defined human demonstrations, and not on non-specific or biased action selection, we conducted an additional negative control test. Following the “control” condition in Topal et al. (2006), we tested Hama's responses in the absence of human demonstration (i.e., no response; Table 1). In "no response" trials, the trainer was stationary for two seconds from the point of returning to the initial posture and making eye contact. If Hama’s responses were influenced by unintentional cues (such as subtle facial and body movements from the demonstrator, experimental assistant, and environmental stimuli, etc.) rather than human demonstrations, she might choose an action from her repertoire of learned actions, even when no action was demonstrated. However, we expected a passive or “no” response if Hama was accurately matching her actions to human demonstrations.

During the test session, six untrained actions that were successfully reproduced in Test 2, the “no response trial,” was randomly incorporated into the session. We performed 10 trials each of the “no response” and each action type.

One session consisted of 14 trials (i.e., two trials of no response and two trials of each of untrained six action types), for a total of 70 trials tested in five sessions. All of her responses were rewarded by food.

Test 4 was conducted by the experimenter (MS) from October 11 to October 13, 2024.

### Study 2: non-simultaneous DAID response in Hama

To control the Clever Hans effect and further validate Hama's ability to perform motor imitation, we implemented a training protocol for non-simultaneous DAID responses. This training followed a conventional DAID paradigm (Topal et al., 2006) where Hama was required to wait until the demonstrator completed his demonstration and a “Go!” vocal cue was presented before initiating an imitation response.

#### Non-simultaneous DAID training phase

We selected six actions (i.e., Spin, Kiss, Stand, Leg, Nod, and Head) that Hama successfully reproduced in Test 2 as our target actions. Initially, we trained Hama to inhibit simultaneous responses. This involved presenting a stationary signal (i.e., showing both palms to Hama and saying “Oboete,” meaning “Remember” in Japanese), followed by the demonstrator returning to the initial posture and beginning the demonstration. Hama received food rewards for remaining stationary until the demonstration was completed. Each training session lasted 5 min, with 1–3 sessions per day from September 1 to 30, 2024.

Next, we trained Hama to reproduce the demonstrated actions after “Go!” vocal cue (i.e., the imitation cue). If Hama failed to perform the desired actions, the same action was demonstrated again, allowing for simultaneous DAID responses. We waited for Hama to generate spontaneous correct responses while avoiding other physical guidance. Each training session lasted five minites, with 1–3 sessions per day from October 1 to 30, 2024. Through this training, Hama successfully reproduced the ideal actions in first session for Kiss, Spin, Stand, and Leg. However, Hama couldn’t reproduce the ideal actions for Nod and Head within the training period.

We temporarily suspended our non-simultaneous DAID training but restarted training from January 25 to 30, 2025.

#### Non-simultaneous DAID test phase

i) Test 5: Non-simultaneous DAID response test

We tested the performance accuracy of Hama’s non-simultaneous DAID responses for the four actions successfully learned during training phase. We conducted a total of 40 trials, with 10 trials for each action type. Each 8-trial session consisted of 2 trials of each action, presented in a randomized order, ensuring no consecutive repetitions of the same action. Hama received food rewards for all her responses. The experimenter MS conducted the testing from January 31 to February 4, 2025.

ii) Test 6: Visual blocking test (control trials for Clever-Hans effect)

To eliminate the Clever Hans effect on Hama's DAID response, we conducted a test with a partition blocking visual contact between Hama and the demonstrator after the demonstration. From February 6 to February 8, we familiarized Hama with a plastic cardboard board (70 cm × 90 cm) by initially placing it within her visual field during DAID training. Subsequently, we familiarized her with the board movement by slightly shifting it after each demonstration. Once Hama's delayed DAID responses were unaffected by the board’s movement, we proceeded with the test.

During the test, a plastic platform was placed 1 m across from the Hama’s starting position, allowing the board to slide. The demonstrator stood behind the platform, and an assistant positioned to the demonstrator’s left operated the board. An additional board (50 cm × 125 cm) concealed the assistant from Hama (Fig. 5: Before demonstration). Immediately after the demonstration, the assistant slid the board toward the demonstrator, completely blocking Hama’s view (Fig. 5: After demonstration). With visual contact blocked, the demonstrator gave “Go!” signal, initiating Hama's DAID response. The assistant confirmed Hama's action through a peephole on the board and notified the demonstrator. The number and order of trials were identical to Test 5. Hama received food rewards for all responses. The experimenter MS conducted the test from February 9 to 12, 2025.

iii) Test 7: Non-simultaneous DAID test for untrained actions

We tested whether Hama’s non-simultaneous DAID responses generalized to untrained actions that were not used in non-simultaneous DAID paradigm. The target actions were four of the 12 actions from the simultaneous DAID test that were not used in Tests 5 and 6. During delayed response training, Hama successfully reproduced the whole-body actions (i.e., Kiss, Spin, Stand, Leg) but struggled with small actions (i.e., Nod, Head). Therefore, we selected two whole-body actions (Hand, Down) and two facial actions (Mouth, Tongue) for this test.

We conducted 10 trials for each untrained action. Each session consisted of 10 trials, including the four untrained actions and six trained actions. The order of actions was randomized with the following constraints. To reinforce that these were DAID sessions, the first action in each test session was a trained action. Additionally, to reduce Hama’s cognitive load from consecutive novel stimuli, no more than three untrained actions were presented consecutively. To minimize learning effects during the testing, we conducted a maximum of one test session per day. The experimenter MS conducted this test from February 17 to 28, 2025.

### Behavior coding

In Study 1 (Test 1–4), we allowed Hama to respond immediately after the trainer's demonstration. We defined the first behavior that Hama exhibited within two seconds of the human demonstration starting as her response. When Hama completed her action, the human demonstrator would say “Yoshi (meaning “Good” in Japanese)” and provide a food reward. This marked the end of the trial. After giving the food reward, the demonstrator returned to the initial position and posture, immediately started the next demonstration, and moved on to the next trial. If Hama did not take any action or remained passive within two seconds after the human demonstration, the trial was also terminated with the “Yoshi” command and a food reward. In the “no response” trials in Test 4, the demonstrator would take the initial position and posture remain still for two seconds. The trial ended after two seconds in the same manner, regardless of any actions Hama produced.

In Study 2 (Test 5–7), Hama’s response was defined as the first behavior performed after the human demonstrator completed the demonstration and gave the “Go!” command. As in all tests, upon completion of Hama’s action, the demonstrator said “Yoshi (meaning “Good” in Japanese)” and provided a food reward, ending the trial. The demonstrator then returned to the initial position and posture, immediately began the next demonstration, and proceeded to the next trial. If Hama remained passive or took no action within two seconds after the human demonstration, the trial was also terminated with the “Yoshi” command and a food reward.

For each trial, Hama’s behavioral response was coded as a binomial variable of “correct” or “incorrect” response based on the behavioral definitions determined in advance (Tables 1). The demonstrators (MS and HK) coded Hama’s response on-site. In Tests 5–6 for Study 2, any response initiated before the “Go!” cue was coded as “incorrect”. However, in Test 7, which targeted untrained actions, we anticipated potential difficulties with delayed responses. Therefore, if Hama initiated a response before the “Go!” cue, she was allowed to return to her initial posture and retry. In this case, if Hama performed the correct response on the second “retried” trial, we also coded these responses as “correct”. If she failed to delay her response twice consecutively, the response was then coded as “incorrect”.

All test sessions were video recorded. In Study 1 (Tests 1–4), the video was taken by a staff member not involved in the study, ensuring they could not anticipate Hama’s expected responses. The camera was positioned at a diagonal angle to Hama’s left, allowing both Hama's face and the trainer's side profile to be visible. During the “No” demonstration trial of Test 4, the staff recording the video was intentionally misled. They were provided with false information suggesting that Hama would perform a particular action. These deceptive trials were employed to minimize the potential influence of the staff member’s expectations on Hama's behavior.

In Study 2 (Tests 5–7), a wider-angle video was required, so we used an unattended GoPro camera fixed at the experimental location. In Tests 5 and 6, which involved large actions, the camera was positioned behind the trainer to capture the movements of the trainer, assistant, and Hama. In Test 7, which included facial actions, the camera was positioned at the same angle as in Tests 1–4 to record the trainer's and Hama's faces and body actions.

### Data analysis

To assess inter-observer agreement, 1/3 of videos were cross coded by a third person who was naïve to this study. Cohen’s Kappa coefficient between the coders was 0.944, suggesting that the reliability can be considered as very good.

Probability of correct response (%) was calculated based on 10 trials for each action type. A binomial test was used to evaluate the number of correct responses against incorrect responses for each action. Since a total of 10 action types were given by human demonstrator during DAID training and test sessions (Test 1 and 2), theoretical probability of correct rate (i.e., chance level) was set at 1/10 (i.e., 10%), which was a conservative criterion considering Hama had a repertoire of more than 50 trained actions. In Study 2, the chance level was set at 10%, consistent with the 10 target actions used in the simultaneous DAID of Study 1.

For Test 2 and Test 3, differences in the number of correct responses between five sessions in the first half and five sessions in the second half were assessed using Chi-Square Test to evaluate changes in Hama’s performance during the test session. We assessed differences in the number of correct responses between Test 5 and Test 6 using a Chi-Square Test to evaluate the influence of unintentional cueing. For Test 3, differences in the number of correct responses between body-oriented and object-manipulation actions was assessed using Fisher’s Exact Test.

All tests were two tailed and set at an alpha = 0.05.

We have deposited the raw data of behavior coding as supplemental materials.

### Ethical notes

The present study was conducted in accordance with the institutional ethics at the Kinosaki Marine World, a member of Japanese Association of Zoos and Aquariums (JAZA). The experimental design of this study adhered to the animal welfare regulations of JAZA and the guidelines for the treatment of animals in behavioral research and teaching by the Animal Behavior Society and Association for the Study of Animal Behavior (Buchanan et al. 2020), and was approved by the ethical management branch of the facility (October 18, 2023). Steller sea lions at the facility receive meticulous daily care, including necessary clinical veterinary treatment provided monthly, to ensure their health and well-being.

## Results

### Test 1: the trained actions and test 2: the untrained actions

**Fig. 2:**
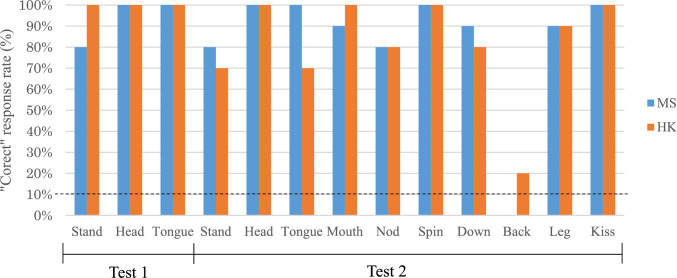
Hama’s Correct” response rate (%) for three trained actions in Test 1 and seven untrained actions in Test 2. (*Note*: The dot line indicates the chance level)

Figure 2 shows Hama’s “correct” response rate (%) for three trained actions in Test 1 (i.e., Stand, Head, and Tongue) and for seven untrained actions in Test 2 (i.e., Mouth, Nod, Spin, Down, Turn, Leg, and Kiss). Hama’s overall correct response rate for Test 1 and Test 2 was 93.3% (28/30) and 83% (83/100) for the first experimenter (MS), and 100% (30/30) and 81% (81/100) for the second experimenter (HK), respectively. Binomial tests for each action revealed that Hama’s actions significantly matched the human demonstration more than the chance level in all actions (*p* < 0.001) except for Back. This pattern was consistent across test trials with both the first experimenter (MS) and the second experimenter (HK).

Hama produced “incorrect” actions in 38 trials (14.6%) of the total trials. These actions were classified either as “incomplete” actions (1 for MS), as another of ten trained actions (15 for MS and 17 for HK), as other actions in her prior learning repertoire (1 for MS), or as no response (2 for MS and 2 for HK).

Table 2 shows Hama’s response for each session in Test 2. Hama’s correct response rate for the untrained actions in the first session was 85.7% (6/7) in demonstrations by MS and 57.1% (4/7) in those by HK. In the first half of the test (sessions 1–5), Hama correctly responded to 86.0% (43/50) in demonstrations by MS and 80.0% (40/50) in those by HK, respectively. In the second half of the test (sessions 6–10), Hama correctly responded to 80.0% (40/50) in demonstrations by MS and 82.0% (41/50) in those by HK, respectively. A chi-square test revealed no significant difference between the number of correct responses in the first and second halves of the test (X^2^ = 0.14, *p* = 0.713).

**Table 2:** Hama’s response in each session for each action type in Test 2

Sessions	1		2		3		4		5		6		7		8		9		10	
Demonstrator	MS	HK	MS	HK	MS	HK	MS	HK	MS	HK	MS	HK	MS	HK	MS	HK	MS	HK	MS	HK
*Actions*																				
Stand	C	C	I	C	C	I	C	I	C	C	C	I	C	C	C	C	C	C	I	C
Head	C	C	C	C	C	C	C	C	C	C	C	C	C	C	C	C	C	C	C	C
Tongue	C	C	C	C	C	C	C	I	C	C	C	I	C	C	C	I	C	C	C	C
Mouth	C	C	C	C	C	C	C	C	C	C	C	C	I	C	C	C	C	C	C	C
Nod	C	I	C	C	C	C	C	C	C	C	C	C	C	I	C	C	I	C	I	C
Spin	C	C	C	C	C	C	C	C	C	C	C	C	C	C	C	C	C	C	C	C
Down	C	I	C	C	C	C	I	I	C	C	C	C	C	C	C	C	C	C	C	C
Back	I	I	I	I	I	I	I	C	I	C	I	I	I	I	I	I	I	I	I	I
Leg	C	C	C	C	C	C	C	C	C	I	C	C	I	C	C	C	C	C	C	C
Kiss	C	C	C	C	C	C	C	C	C	C	C	C	C	C	C	C	C	C	C	C

“C” indicates “Correct” response, and “I” indicates “Incorrect” response

### Test 3: the novel actions not in the learning repertoire

**Fig. 3:**
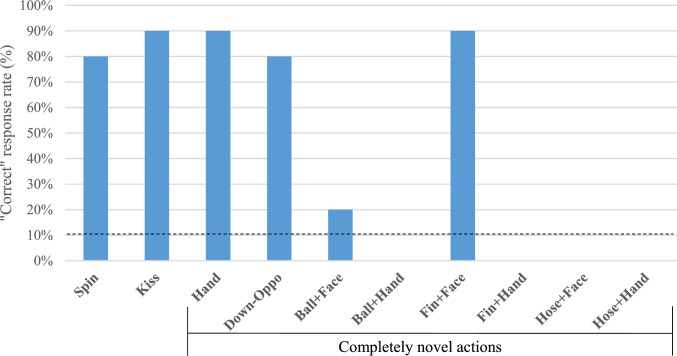
Hama’s“Correct” response rate (%) for novel actions not in her learning repertoire (Test 3). (*Note*: The dot line indicates the chance level)

Figure 3 shows Hama’s “correct” response rate (%) for eight novel actions including two body-oriented (i.e., Hand and Down-Oppo) and six manipulative actions (i.e., Ball-Face, Ball-Hand, Fin-Face, Fin-Hand, Hose-Face, and Hose-Hand) in Test 3. Both of the body-oriented actions had significantly higher correct rates (90% and 80%) than the chance level (binomial test, all *p* < 0.001). On the other hand, five out of the six object manipulative actions did not differ in correct rate (all 0% except for 20% in Ball-Face) from the chance level (binomial test, *p* = 0.613, except for *p* = 0.26 in Ball-Face). Only Fin-Face action had a significantly higher correct rate (90%) than the chance level (binomial test, *p* < 0.001).

The number of correct and incorrect responses in the body-oriented actions was 17 and 3, respectively, while those in the object-manipulative actions were 11 and 49, respectively. Fisher’s Exact tests revealed that Hama’s correct response rate of the body-oriented actions was significantly higher than those of the object-manipulative actions (*p* < 0.001).

Hama produced “incorrect” actions in total of 54 trials (54.5%), including data from Spin and Kiss commands. These actions were classified either as “incomplete” actions (N = 3), as another of eight novel actions (N = 35), as other actions in her prior learning repertoire (N = 6), or as other irrelevant behaviors (e.g., no response and sniffing on the ground, N = 10). Hama often touched the objects with her muzzle and never used a fore flipper. She chose the Fin-Face action the most frequently (N = 33) when demonstrated the object manipulative action by the experimenter.

Table 3 summarizes Hama’s responses for each session in Test 3. In the two novel body-oriented actions (i.e. Hand and Down-Oppo), Hama correctly responded to the Hand action but incorrectly responded to Down-Oppo in the first session. For six object-manipulative actions, Hama correctly responded to only the Fin-Face action sequence, resulting in a correct response rate of 16.7% (1/6) in the first session. In the first half of the test (sessions 1–5), Hama achieved a 90.0% (9/10) success rate in novel body-oriented actions. Conversely, Hama’s correct response rate in this period was 13.8% (4/29) of object-manipulative actions. In the second half of the test (sessions 6–10), Hama correctly performed 80.0% (8/10) of the novel body-oriented actions and 20.0% (6/30) of object-manipulative actions. Despite the incorrect response to Down-Oppo action being reinforced with food rewards in the first session, Hama’s correct response rate to this action type was high (88.9%; 8/9) in all subsequent trials of sessions 2–10. A chi-square test revealed no significant difference between the number of correct responses in the first and second halves of the test (X^2^ = 0.04, *p* = 0.849).

**Table 3:** Hama’s response in each session for each action type in Test 3

Sessions	1	2	3	4	5	6	7	8	9	10
Demonstrator	MS	MS	MS	MS	MS	MS	MS	MS	MS	MS
*Actions*										
Spin	–	C	C	C	C	C	C	C	I	C
Kiss	C	C	C	C	C	C	I	C	C	C
Hand	C	C	C	C	C	C	C	C	I	C
Down-Oppo	I	C	C	C	C	I	C	C	C	C
Ball + Face	I	I	I	I	I	I	I	I	C	I
Ball + Hand	I	I	I	I	I	I	I	I	I	I
Fin + Face	C	I	C	C	C	C	C	C	C	C
Fin + Hand	I	I	I	I	I	I	I	I	I	I
Hose + Face	I	I	I	I	I	I	I	I	I	I
Hose + Hand	I	I	–	I	I	I	I	I	I	I

“C” indicates “Correct” response, “I” indicates “Incorrect” response, and “–” indicates “No conducted (i.e., procedural error)”

### Test 4: no demonstration control

**Fig. 4:**
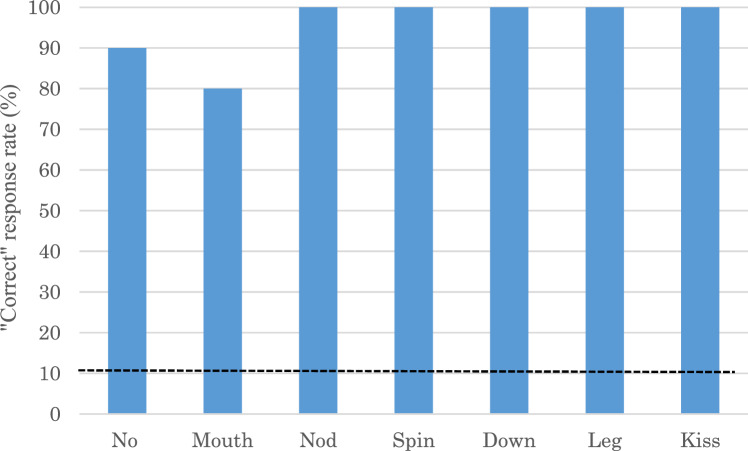
Hama’s “Correct” response rate (%) in Test 4 (including “No demonstration” trials). (*Note*: The dot line indicates the chance level)

**Fig. 5:**
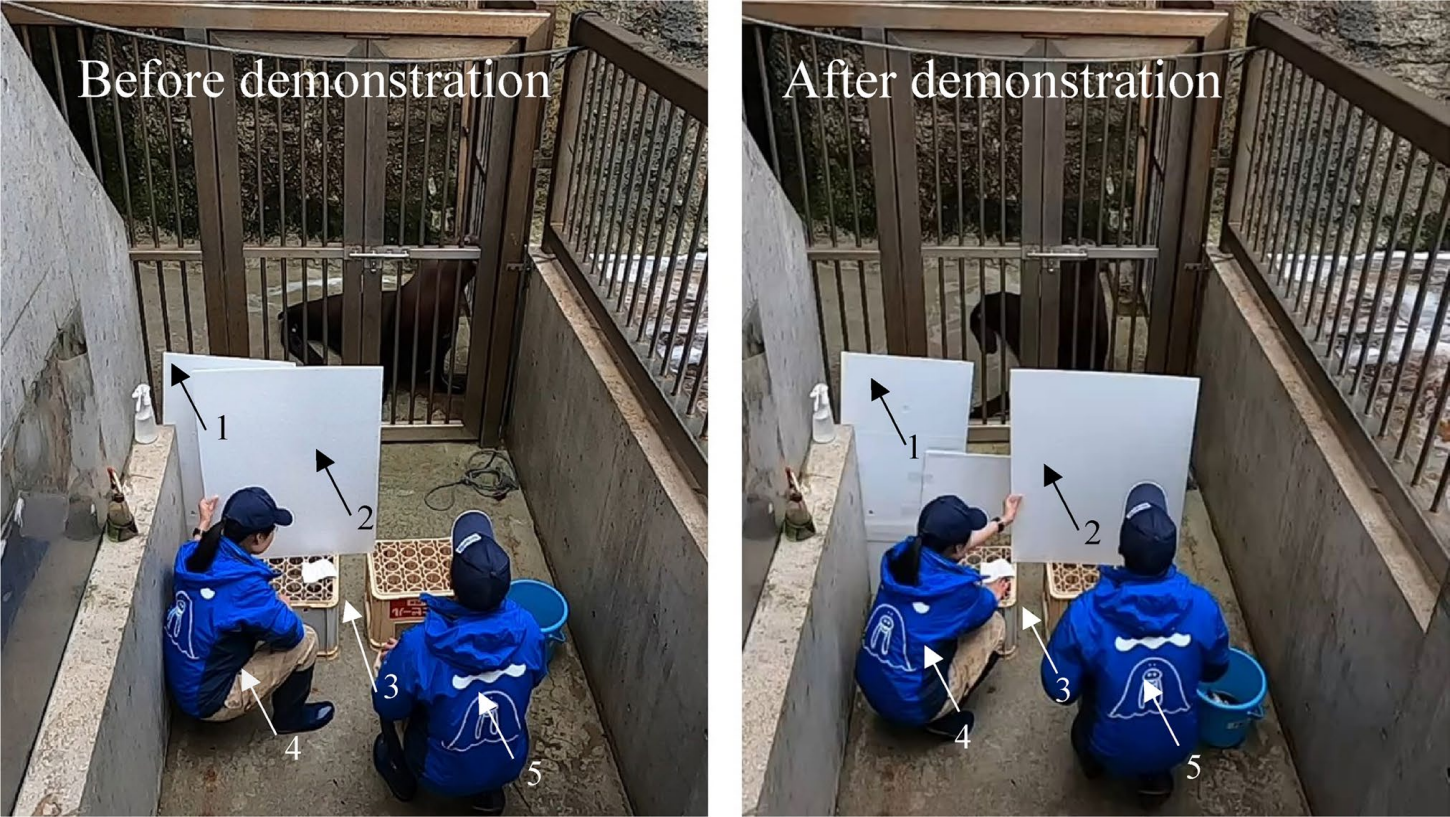
Arrangement of the materials in the experimental location (Test 6). (*Note*: Arrow 1 indicates the board that was placed in front of the assistant; arrow 2, the board in front of the demonstrator; arrow 3, the plastic platform; arrow 4, the assistant; and arrow 5, the demonstrator)

Figure 4 shows Hama’s “correct” response rate (%) for six actions (i.e. Mouth, Nod, Spin, Down, Turn, Leg, and Kiss) and control trials (i.e., No demonstration) in Test 4. Notably, Hama performed no action (passive) in response to human passive demonstrations in nine out of 10 control (No-demonstration) trials. This indicates that no-demonstration control trials had a significantly higher “correct” rate (90%) than chance level (binomial test, *p* < 0.001). Hama “incorrectly” responded with the “Tongue” action only once in a no-demonstration trial. All six untrained actions also had significantly higher correct rates (overall 96.7%) than the chance level (binomial test, all *p* < 0.001). Hama responded incorrectly with “Tongue” and “No” actions during two trials of “Mouth” demonstrations, respectively (Fig. 5).

### Test 5: non-simultaneous DAID test and test 6: visual blocking test

**Fig. 6:**
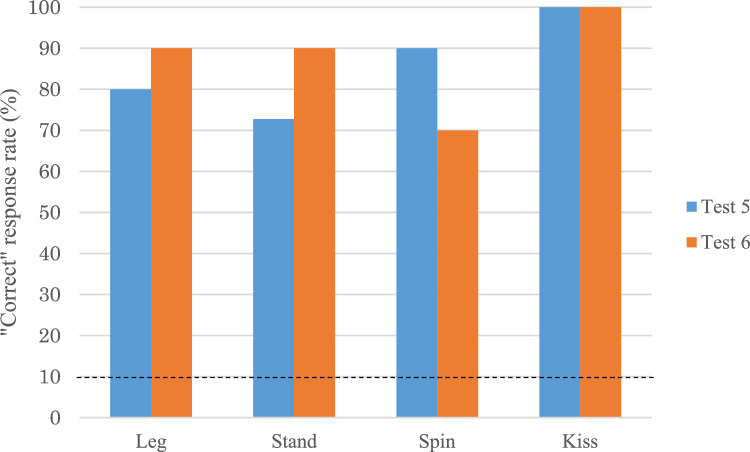
Hama’s “Correct” response rate (%) in “Non-simultaneous DAID response”. (*Note*: The dot line indicates the chance level)

Figure 6 shows Hama’s “correct” response rate (%) for four actions (i.e. Stand, Spin, Leg, and Kiss) in Test 5 and Test 6. All actions had significantly higher “correct” rates than the chance level (binomial test, all *p* < 0.001), even when Hama’s visual contact with the demonstrator was completely blocked. A chi-square test revealed no significant difference between the number of correct responses in Test 5 and Test 6 (X^2^ = 0.08, *p* = 0.78), indicating that Hama’s performance was not significantly affected by inadvertent cues from the experimenter. Hama produced a total of six “incorrect” actions (14.6%) in Test 5 and five “incorrect” trials (12.5%) in Test 6. In Test 5, “incorrect” actions were categorized as either “incomplete” actions (N = 2) or as one of the other three tested actions (N = 4).

### Test 7: non-simultaneous DAID test for untrained actions

All four untrained actions did not differ in correct rate (all 0% except for 10% in Hand) from the chance level (binomial test, *p* = 0.349, except for *p* = 0.387 in Hand). In untrained actions, there were three occasions when Hama could not wait for her response until the “Go!” cue after the demonstration to respond, but she never responded correctly on the second attempt.

Hama produced “incorrect” actions in total of 43 trials (45.3%), including data from trained actions (i.e., Spin and Kiss). These actions were classified either as “incomplete” actions (N = 1), as another of three untrained actions (N = 3), as other trained actions that were trained in non-simultaneous DAID training phase (N = 35), as other actions in the prior learning repertoire (N = 2), or as no response (N = 2).

Table 4 summarizes Hama’s responses to four untrained actions. Hama responded to actions involving foreflipper motion (i.e., Hand, Kiss and waving foreflipper) in 9/10 Hand trials (90%; including 2 trials that Hama could not wait and retried). For Down trials, 8/10 responses (80%; including 1 trial that Hama could not wait and retried.) involved a large downward head motion (i.e., Leg). Furthermore, Hama consistently responded with a Nod action to all trials of Mouth and Head, actions that specifically involve facial movement.

**Table 4:** Hama’s non-simultaneous response for untrained actions

	Hama's action								
	Hand	Down	Mouth	Tongue	Nod	Kiss	Leg	Waving hand	Incomplete
*Human action*									
Hand	1				1	6		2	
Down					1		8		1
Mouth					10				
Tongue					10				

The number in each cell indicates the number of observed responses

## Discussion

The present study explored whether a well-socialized Steller sea lion named Hama could reproduce similar actions with human demonstrations using the DAID paradigm. Study 1 tested Hama’s simultaneous DAID performance. We found that Hama successfully acquired the action-matching ability for human actions, not only with the trained actions but also with the untrained actions (see Test 1 and Test 2). Hama’s DAID performance was stable regardless of the familiarity of human demonstrators (see Test 1 and Test 2). No-demonstration control trials offered partial evidence for negative control (Test 4). Moreover, Hama could produce similar body-oriented action sequences for completely novel actions that were not included in her prior learning repertoire. However, Hama could not generalize her imitative behavior to novel action sequences for object-manipulative actions (Test 3).

In Study 1, Hama learned quickly to perform DAID responses to three actions (Up, Head, Tongue) within the first four training sessions. Her high correct response rate in Test 1 confirmed successful acquisition of the DAID skills for trained actions. These data suggest that Hama might recognize similarity between the observed and her own behavior at an early stage of the training. Furthermore, Hama performed well on six untrained actions (Mouth, Nod, Spin, Down, Leg, and Kiss) in Test 2, suggesting that she successfully generalized her ability to match the human demonstrator's actions with her own ones. Hama's DAID performance was exceptional from the first session, suggesting that her imitative responses did not stem from learning effects from repeated test trials. Even actions that were reinforced as incorrect in the first session were correct in the subsequent session, indicating that the learning effect from reinforcing all trials of a test session is relatively small. Additionally, there was no difference in the correct response rate between the first and second halves of the test session, suggesting that Hama acquired generalized imitative skills through prior DAID training and applied them to untrained actions rather than learning effects during the test period. While these action types were operant-conditioned earlier, Hama’s actions were never reinforced with similar action demonstrations by humans. Thus, DAID training facilitates her to use the actions of a human demonstrator as cues to match her own actions, instead of hand signals and vocal commands learned previously.

Hama successfully matched her actions not only to the action demonstrations shown by the first trainer introducing DAID training but also to those presented by the novel trainer. This ruled out the possibility of unconscious cueing by the particular familiar human demonstrator (i.e., the Clever Hans effect), providing additional evidence of Hama transferring the copying rule to other situations and contexts. Similar generalization to other demonstrators has been reported in studies with dogs (Topál et al. 2006). Trials without demonstration in Test 4 further eliminated the possibility of unintentional cueing from the demonstrator or other environmental factors on Hama’s responses. In the absence of demonstration, Hama remained passive and responded “no response” in most trials, suggesting that Hama’s imitative responses were unlikely to be due to subtle unintentional cues other than the demonstrator's demonstration. These results were comparable with the dog in Topál et al. (2006), confirming that the prior DAID training phase successfully induced Hama’s imitative ability to match between her own action and human demonstration.

Although Hama's overall performance in response to human demonstrations in Test 1 and Test 2 was significantly better than chance, she could not transfer her DAID response to the "Back" command. Hama made incorrect responses in 80% (16/20 trials) by assuming Down posture. This might be confused with Back posture because both involve lowering the head and abdomen. Notably, Hama usually transitions from Down to Back posture. Therefore, Hama's incorrect Down responses might be interpreted as an incomplete or partial Back posture. This reluctance to lie on her back can be explained by the social status of Hama in the group and the location of the cage. Hama's subordinate position within the group might influence negatively her willingness to expose her abdomen to other dominant individuals, particularly in the test area where she is in close proximity to other group members. In fact, Hama tended to be reluctant to expose her belly in the presence of other individuals, even when the trainer requested her to do so. Previous data suggested that Hama successfully performed the Back posture in over 90% of the trials when instructed by voice command in the isolated training areas away from the group-living enclosure (Sasaki et al. 2022).

More interestingly, Hama showed successful transfer of her DAID responses to two types of novel body-oriented actions (i.e., Hand, Down-Oppo) that had never been learned previously. Hama could perform correctly from the beginning of the session, and there was no difference in the correct response rate between the first and second halves of the test period, confirming that Hama was able to generalize the basic rules of DAID to completely novel actions. Generalization of the copying concept to novel actions has been reported only in a very limited species including chimpanzees (e.g., Hribar et al. 2014), dogs (e.g., Topal et al. 2006), gray parrots (e.g., Pepperberg 1988), dolphins (e.g., Herman 2002), and orcas (e.g., Zamorano-Abramson et al. 2013). These prior researches also suggest DAID imitation was limited to specific, highly encultured individuals. Generalized DAID imitation of body-oriented (intransitive) actions was found in even fewer species, such as cetaceans (Zamorano-Abramson et al. 2023; discussed fully in the next paragraph). Hama's impressive performance in imitating body-oriented actions suggests that Steller sea lions can develop social learning skills for mapping human body parts and motor patterns from novel gestural cues and generating their own corresponding behaviors.

On the other hand, Hama had difficulty matching object-manipulative actions (i.e., transitive actions) by a human demonstrator with her own ones. She achieved a significant success rate only in the "Fin-Face" action. However, this likely resulted from Hama simply touching a swim fin with her muzzle in most trials, regardless of the different action sequences towards objects being demonstrated by a human. This could be interpreted as meaning that when Hama lay down to touch an object on the floor, she contacted the fin because it was closest to her snout (the object's location remained fixed throughout all trials in Test 3). There are two possible explanations for Hama's poor performance on object-related action imitation tasks. Firstly, Hama might have insufficient cognitive processing ability to grasp the complex relationship between object variations and the corresponding behavioral changes of the human demonstrator. Secondly, Hama has less prior experience being trained on object-related actions compared to body-oriented actions. Hama's training history differed from that of Philip, an assistance dog for people with disabilities, which successfully copied human demonstrations of moving a specific object from one location to another (Topál et al., 2006).

Hama’s difficulty in imitating object-related actions may be due to the methodology of the current study. In this study, body-oriented actions were trained on a small set of actions before testing with untrained actions. On the other hand, object manipulative actions were not trained using the DAID method, except for familiarization with the target objects. This was because Test 3 was intended to test whether the previous DAID rules could be transferred for completely novel actions for Hama. Similarly, if we had conducted generalization training with a small set of object-manipulative actions, Hama's test performance in novel object manipulative actions might have been improved.

Hama’s greater success with body-oriented actions highlights species-specific cognitive traits in how they develop motor imitation within the DAID paradigm. Prior research shows successful transfer of DAID imitation to novel object-oriented actions (i.e., transitive actions) in chimpanzees, dogs, and cats (Topál et al., 2006; Huber et al., 2009; Fugazza et al., 2021). Conversely, captive cetaceans, similar to Hama in this study, have succeeded in matching actions of a demonstrator in body-oriented actions (i.e., intransitive actions) (Zamorano-Abramson et al., 2013; Zamorano-Abramson et al., 2018). The discrepancy in the pattern of motor imitation cannot be explained solely by similarity between human body structure and their own, suggesting a complex link between processing human actions and reproducing them within the DAID framework. Copying intransitive actions seems to be cognitively more challenging than object-related transitive actions (Tennie et al., 2009; Heyes and Ray 2000; Zentall 2003, 2011). This is because reproducing body-related behaviors relies entirely on observing the demonstrator's actions without the additional cues for action selection provided by objects. Further research is needed to explore how transitivity in motor imitation is affected by body structure, habitat environment, and social cognitive abilities of the species.

While Hama's performance in Study 1 suggests the acquisition of a general rule of action copying in motor imitation tasks, this demonstrator-matching behavior could also be explained by simpler learning mechanisms, such as local enhancement and/or affordance learning (Zentall, 2012). The experimental procedures in Study 1 allowed Hama's simultaneous responses following the human demonstration, potentially reflecting automatic facilitative processes underlying simultaneous imitation (Heyes and Ray, 2000). Furthermore, while Study 1 implemented control conditions such as changing the demonstrator (Test 1, Test 2) or omitting the demonstration (Test 4), these could not completely rule out unintentional cueing from the demonstrator. The immediate response window in these tests potentially allowed Hama to utilize inadvertent visual cues emitted by the demonstrator. In contrast, the non-simultaneous DAID imitation paradigm, standard in dog research, requires a time delay after observing human demonstrations, followed by imitative behavior in response to a generic vocal command (e.g., "Do it!"), thereby demanding a higher cognitive effort. This is because successful matching behavior after a time delay requires retrieving an enduring mental representation of the target action (Zentall and Galef, 1989). Using this standard DAID method, blocking visual information from the demonstrator is possible during the time delay before the voice command, thereby enabling a stricter ruling out of the Clever Hans effect.

In Study 2, we employed a non-simultaneous DAID paradigm to train Hama’s imitative responses with a delay. Hama demonstrated high DAID performance when her response was withheld until the demonstrator's action was complete and then cued with the “Go” command (Test 5), suggesting her ability to produce imitative behavior using the standard DAID method and perform demonstrator-matching with a generic voice signal. Notably, Hama's DAID performance remained excellent even when visual contact with the demonstrator was blocked after the demonstration (Test 6), strongly mitigating concerns about the Clever Hans effect. However, Hama could not reproduce small or untrained actions in the non-simultaneous DAID (Test 6 and Test 7).

In the first session of non-simultaneous DAID training, Hama rapidly learned to reproduce non-simultaneous imitation of four actions (Stand, Spin, Leg, and Kiss) following the introduction of a voice command for a stationary action. Hama exhibited high non-simultaneous DAID performance in Test 5, and successfully reproduced the ideal actions even under visual occlusion in Test 6–7. These results suggest that Hama formed enduring mental representations of human actions and responded correctly to the abstract concept of imitation conveyed by the generic voice command “Go”, demonstrating her generalized imitative ability. Furthermore, Hama's consistent performance under visual occlusion suggests her ability to maintain action memory despite interfering stimuli, and minimizes the influence of unintentional demonstrator cues during imitation instructions. These findings indicate that Hama's DAID responses cannot be fully attributed to local enhancement and/or affordance learning, and provide an evidence that Hama can accurately reproduce the human actions using only human demonstrations as cues. To the best of our knowledge, no experimental control for the Clever Hans effect under visual occlusion conditions has been conducted in previous DAID studies. Therefore, Hama's results in the present study provide stronger evidence for motor imitation ability.

Hama's non-simultaneous DAID learning varied by action types, with facial actions (i.e., Nod and Head) failing to be imitated during training. There are two possible reasons why Hama could not reproduce the small actions. One potential explanation is that suppressing simultaneous imitative responses to small facial movements is more challenging than for dynamic, whole-body movements. Whole-body movements require preparatory actions and a longer completion time, facilitating delayed responses. Conversely, facial expressions are often imitated instantaneously, potentially due to an automatic response mechanism (Palagi et al., 2015). Another potential explanation is that Hama experienced difficulty in accessing persistent mental representation for small-scale actions. However, research has shown that killer whales can accurately reproduce tongue protrusion and head movements in deferred DAID paradigms, indicating their ability to retrieve enduring mental representations for such actions (Zamorano-Abramson et al., 2023). Although training was intentionally brief to minimize overtraining in the present study, extended training focusing on response suppression for facial movements might yield delayed imitation. While species differences could contribute to these contrasting results, comprehensive comparative studies involving a larger sample size and diverse species are needed to elucidate this possibility.

Hama was unable to reproduce untrained actions in non-simultaneous DAID. However, Hama’s responses indicated partial matching to the demonstrator’s actions, with consistency in either body part or action sequence (see Table 4). This suggests that Hama retained incomplete mental representations of the observed actions. Given that great apes, dogs, and cetaceans demonstrate strong deferred DAID performance with extended delays (Huber et al., 2009; Fugazza and Miklósi, 2014; Zamorano-Abramson et al., 2023), Hama’s developing non-simultaneous DAID skills warrant further detailed investigation in future studies.

The extent to which Hama's DAID imitation can be replicated by other individuals and extended to the common population of sea lions is still unclear. Breeding and training large pinnipeds is not easy, but future research with a larger sample size is necessary to identify the individual and social factors influencing social learning. This could include individual characteristics such as individual rearing history, socialization with humans, and learning repertoires. Ecological factors are also important in understanding the underlying mechanisms of social learning skills. Shusterman et al. (1993) proposed that the sea lions' social structure, characterized by very dense and large social groups, may be a significant factor contributing to the development of their sophisticated cognitive abilities, such as understanding of equivalent concepts. The findings of the present study provide evidence that a socialized Steller sea lion possesses the cognitive skill to imitate actions of heterospecific models. This suggests the ability to form abstract concepts of motor imitation in Steller sea lions and extends our knowledge of social learning in the sea lion family.

## Supplementary Information

Below is the link to the electronic supplementary material.

Supplementary file1 (CSV 22 KB)

## Data Availability

No datasets were generated or analysed during the current study.
